# Crystal structure and magnetic properties of LaCa_0.143 (4)_O_0.857 (4)_F_0.143 (4)_Bi_0.857 (4)_S_2_

**DOI:** 10.1107/S2056989016008082

**Published:** 2016-05-24

**Authors:** Rongtie Huang, Hui Zhang, Dong Wang, Chuanbing Cai, Fuqiang Huang

**Affiliations:** a State Key Laboratory of High Performance Ceramics and Superfine Microstructures, Shanghai Institute of Ceramics, Chinese Academy of Sciences, Shanghai 200050, People’s Republic of China; b Department of Physics, Shanghai University, Shanghai 200444, People’s Republic of China

**Keywords:** crystal structure, magnetic properties, disorder

## Abstract

The synthesis, structure, and magnetic properties of lithium dibarium calcium oxide fluoride di­sulfide are reported. LaCa_0.143 (4)_O_0.857 (4)_F_0.143 (4)_Bi_0.857 (4)_S_2_ crystallizes in the tetra­gonal space group *P*4/*nmm*. The structure exhibits disorder of the Ca^2+^ and Bi^3+^ cations, and the O^2−^ and F^−^ anions. The structure is composed of a stacking of [(O,F)_2_La_2_] layers and double [(Bi,Ca)S_2_] layers. Magnetic property measurements indicate a very small magnetization at 300 K and the existence of weak ferromagnetism at 2 K.

## Abstract 2

LaBi_1.859 (4)_Ca_0.141 (4)_O_0.859 (4)_F_0.141 (4)_S_2_ crystallizes in the tetra­gonal space group *P*4/*nmm*. The structure exhibits disorder of the Ca^2+^ and Bi^3+^ cations, and the O^2−^ and F^−^ anions. The structure is composed of a stacking of [(O,F)_2_La_2_] layers and double [(Bi,Ca)S_2_] layers. Magnetic property measurements indicate a very small magnetization at 300 K and the existence of weak ferromagnetism at 2 K.

## Chemical context   

Layered crystal structures seem to be a common stage on which to explore superconductivity (Vershinin *et al.*, 2004 ▸; Kamihara *et al.*, 2008 ▸; Chen *et al.*, 2008 ▸; Fang *et al.*, 2010 ▸). The discovery of [Fe_2_ *An*_2_] (*An* = P, As, S, Se or Te) and [CuO_2_] superconducting layers has opened a new field in physics and chemistry for the exploration of low-dimensional superconductivity. Recently, superconductivity with transition temperatures of 4.5 K was reported in the BiS_2_-based compound Bi_4_O_4_S_3_ (Singh *et al.*, 2012 ▸). Soon after, *Ln*O_1-*x*_F_*x*_BiS_2_ (*Ln* = La, Ce, Pr and Nd), were reported to be superconducting with transition temperatures *T*_c_ of 3–10.6 K (Nagao *et al.*, 2013 ▸; Demura *et al.*, 2013 ▸). The mother BiS_2_-based layered compound *Ae*FBiS_2_ (*Ae* = Ca, Sr or Ba; Lei *et al.*, 2013 ▸; Han *et al.*, 2008 ▸) is isostructural to *Ln*OBiS_2_, with the [*Ln*_2_O_2_]^2−^ layer being replaced by an isocharged [Sr_2_F_2_]^2−^ block. The parent phase of SrFBiS_2_ shows semiconducting behavior, but electron-doped Sr_0.5_La_0.5_FBiS_2_ has a superconducting transition of 2.8 K (Lin *et al.*, 2013 ▸). Herein the synthesis, structure and magnetic properties of LaCa_0.143 (4)_O_0.857 (4)_F_0.143 (4)_Bi_0.857 (4)_S_2_ are reported.

## Structural commentary   

**Figure 1:**
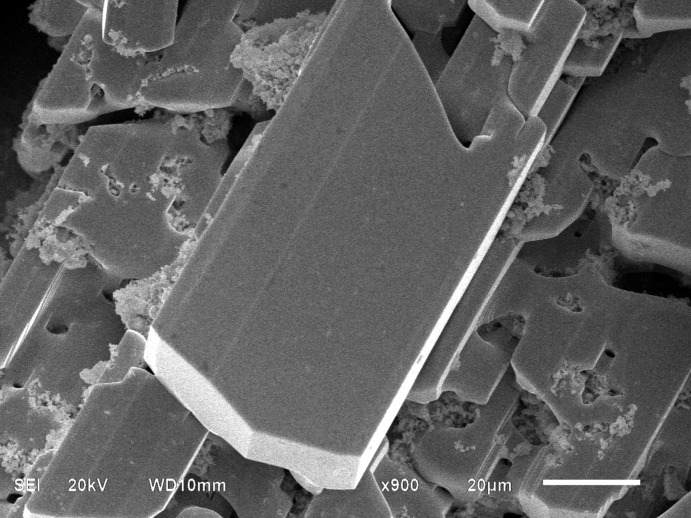
SEM image of LaCa_0.143 (4)_O_0.857 (4)_F_0.143 (4)_Bi_0.857 (4)_S_2_.

**Figure 2:**
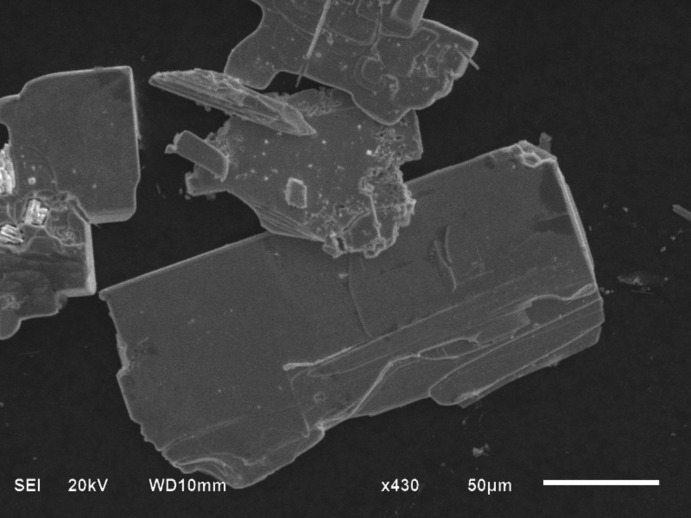
SEM image of LaO_0.6_F_0.4_BiS_2_.

We attempted to prepare the Ca and F double-doped compound La_1−*x*_Ca_*x*_O_1−2*x*_F_2*x*_BiS_2_, but the results indicate the single-crystal composition is LaCa_0.143 (4)_O_0.857 (4)_F_0.143 (4)_Bi_0.857 (4)_S_2_. An SEM image shows thick plate-shaped crystals of LaCa_0.143 (4)_O_0.857 (4)_F_0.143 (4)_Bi_0.857 (4)_S_2_ (Fig. 1 ▸). LaO_1−*x*_F_*x*_BiS_2_ crystals usually show a thin-sheet shape (Fig. 2 ▸). From the EDXS analysis, we obtained the elemental components of La, Ca, Bi, S, F and O. The final composition was obtained by structure refinement (details can be seen in the *Refinement* section).

**Figure 3:**
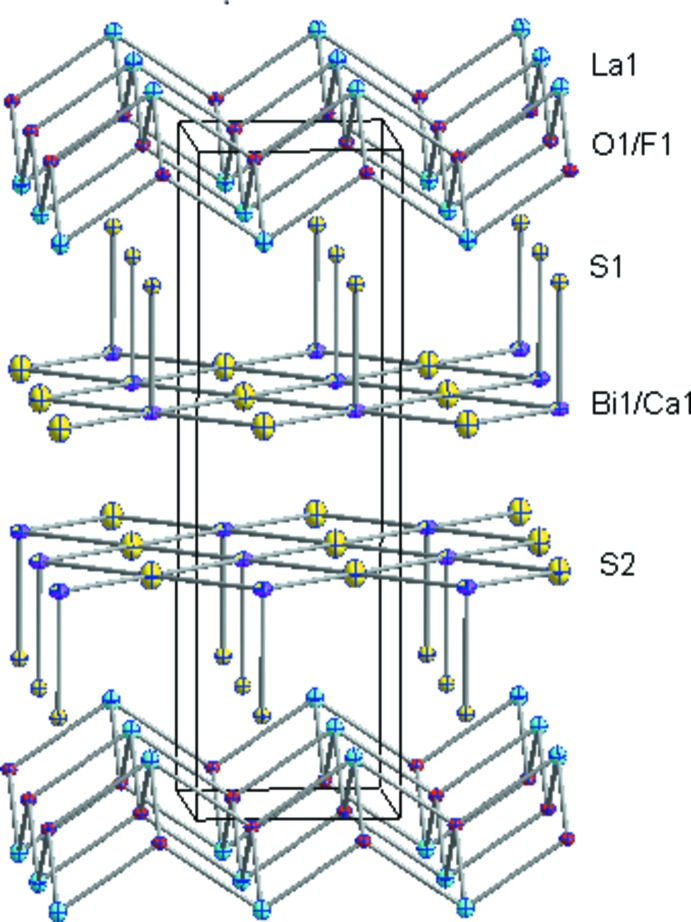
Crystal structure of LaCa_0.143 (4)_O_0.857 (4)_F_0.143 (4)_Bi_0.857 (4)_S_2_, showing [(O,F)_2_La_2_] layers and double [BiS_2_] layers (O/F in red, La in blue, Bi/Ca in pink and S in yellow; 50% probability ellipsoids).

The structure of LaCa_0.143 (4)_O_0.857 (4)_F_0.143 (4)_Bi_0.857 (4)_S_2_, shown in Fig. 3 ▸, is composed of a stacking of [(O,F)_2_La_2_] layers and double [(Bi,Ca)S_2_] layers as in LaO_1−*x*_F_*x*_BiS_2_. The double [(Bi,Ca)S_2_] layers show Bi1/Ca1—S2 distances of 2.8672 (6) Å representing equatorial bonds and Bi1/Ca1—S1 distances of 2.530 (3) Å representing axial bonds; these are a little shorter than the Bi1—S1 distance of 2.87476 (15) and Bi1—S2 distance of 2.530 (6) Å in LaO_1−*x*_F_*x*_BiS_2_. The [(O,F)_2_La_2_] layers exhibit O1/F1—La1 bond lengths of 2.4414 (6) Å and La1—O1/F1—La1 bond angles of 108.08 (2) and 112.29 (4)°, which are close to the La—O/F bond length of 2.4402 (8) Å and La1—O1/F1—La1 bond angles of 107.82 (3) and 112.82 (6)° in LaO_1−*x*_F_*x*_BiS_2_. The ionic radius of Ca^2+^ is 114 pm which is a little shorter than that of 117.2/117 pm for La^3+^/Bi^3+^. The distinct reduced Bi1/Ca1—S2 distances in the title compound reflect the fact that Ca substitutes Bi sites rather than La sites.

## Magnetic property measurements   

**Figure 4:**
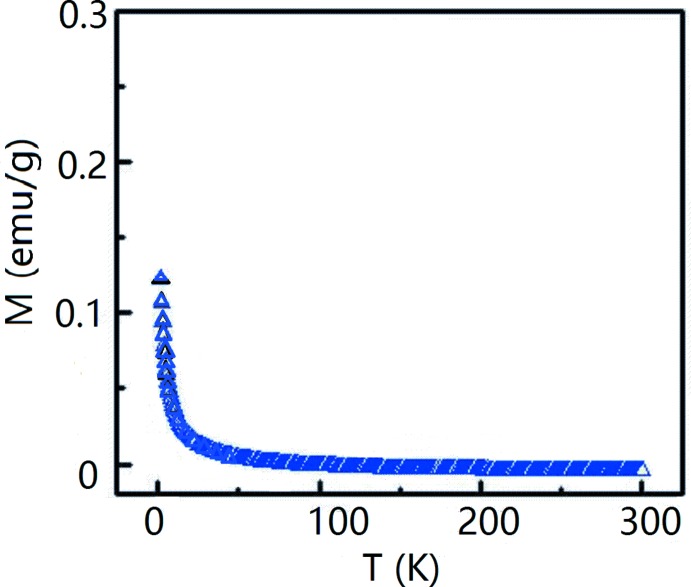
Magnetic moment *versus* temperature for LaCa_0.143 (4)_O_0.857 (4)_F_0.143 (4)_Bi_0.857 (4)_S_2_ under a 1 T field.

**Figure 5:**
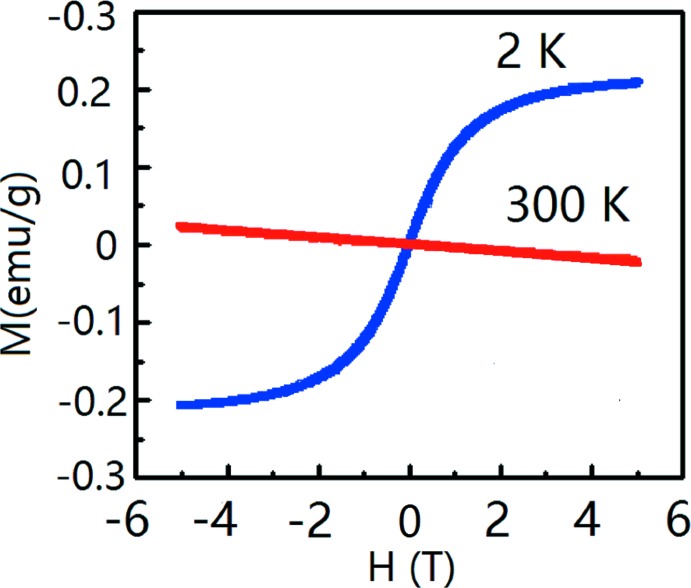
Magnetic moment *versus* field for LaCa_0.143 (4)_O_0.857 (4)_F_0.143 (4)_Bi_0.857 (4)_S_2_ from −5 T to 5 T at 2 K and 300 K.

The magnetization *versus* temperature under a 1 T field for the title compound is given in Fig. 4 ▸. Magnetization *versus* magnetic field is given in Fig. 5 ▸ for fields ranging from −5 to 5 T at 2 K and 300 K. The magnetic properties indicate weak ferromagnetism at 2 K and a very low magnetization at 300 K. The superconducting transition is not observed in the measured temperature range. This might be related to the Ca substitution of the Bi site in the title compound. For superconducting LaO_1−*x*_F_*x*_BiS_2_ crystals, the density of states at the Fermi level is mainly directed by the Bi *p* orbital. [BiS_2_] layers play a vital role in the transport and superconducting properties. The Ca substitution of the Bi site leads to a hole doping which changed the electronic band structure and density of state of LaO_1−*x*_F_*x*_BiS_2_. Another reason might be the reduced F content in LaCa_0.143 (4)_O_0.857 (4)_F_0.143 (4)_Bi_0.857 (4)_S_2_ compared with LaO_1−*x*_F_*x*_BiS_2_.

## Database survey   

*Ln*O_1−*x*_F_*x*_BiS_2_ (*Ln* = La, Ce, Pr and Nd) compounds were reported by Nagao *et al.* (2013 ▸) and Demura *et al.* (2013 ▸). *Ae*FBiS_2_ (*Ae* = Ca, Sr, Ba) (Lei *et al.*, 2013 ▸; Han *et al.*, 2008 ▸) are isostructural to *Ln*OBiS_2_. The doped Sr_0.5_La_0.5_FBiS_2_ (Lin *et al.*, 2013 ▸) is isostructural to *Ae*FBiS_2_.

## Synthesis and crystallization   

LaCa_0.143 (4)_O_0.857 (4)_F_0.143 (4)_Bi_0.857 (4)_S_2_ was prepared using of Bi_2_O_3_, CaF_2_, La_2_S_3_, Bi_2_S_3_ and Bi raw materials. The mixtures with a nominal composition of La_0.85_Ca_0.15_O_0.70_F_0.30_BiS_2_ were ground, pressed into pellets, sealed in an evacuated quartz tube, and heated at 1073 K for 3 d. High-quality single crystals were grown by using KI as the flux. Nominal La_0.85_Ca_0.15_O_0.70_F_0.30_BiS_2_ and KI in the molar ratio of 1:3 were mixed and placed in a quartz tube, which was sealed and heated to 1273 K and kept at this temperature for 1 d, then cooled to room temperature in 10 d. The product was washed with distilled water and acetone, then dried at 353 K for 12 h; finally black plate-shaped crystals were obtained.

The morphology and element compositions were investigated by a scanning electronic microscope equipped with an energy dispersive X-ray spectroscopy (EDXS, Oxford Instruments). The EDXS shows the atom % ratio for S:Ca:La:Bi to be 48.23: 6.94: 24.36: 20.48. O and F could not be determined precisely. Magnetic properties were measured on a multifunctional physical properties measurement system (PPMS, Quantum Design).

## Refinement   

Crystal data, data collection and structure refinement details are summarized in Table 1 ▸. The La, Bi, S and O atoms were located in difference maps and their positions were freely refined. Ca was assumed at La sites at first, but the refinement show no reducing occupancy of La. The partial occupancy of Bi indicates a mixed occupancy with Ca. Ca and Bi were refined together later. EDXS measurements could not determine occupancies of O and F precisely. If the occupancies of F and O are refined together, the obtained composition is La_2_Bi_1.859 (4)_Ca_0.141 (4)_O_0.48 (14)_F_0.52 (14)_S_4_ with high standard errors for O and F. In order to keep charge neutrality, the occupancy of F was fixed to be the same as Ca so the final composition of LaCa_0.143 (4)_O_0.857 (4)_F_0.143 (4)_Bi_0.857 (4)_S_2_ was obtained.

## Supplementary Material

Crystal structure: contains datablock(s) I, New_Global_Publ_Block. DOI: 10.1107/S2056989016008082/pj2030sup1.cif

Structure factors: contains datablock(s) I. DOI: 10.1107/S2056989016008082/pj2030Isup2.hkl

CCDC reference: 1480636

Additional supporting information:  crystallographic information; 3D view; checkCIF report

## Figures and Tables

**Table 1:** Experimental details

Crystal data	
Chemical formula	LaCa_0.143 (4)_O_0.857 (4)_F_0.143 (4)_Bi_0.857 (4)_S_2_
*M*_r_	404.26
Crystal system, space group	Tetragonal, *P*4/*n* *m* *m*
Temperature (K)	300
*a*, *c* (Å)	4.0548 (9), 13.370 (3)
*V* (Å^3^)	219.82 (11)
*Z*	2
Radiation type	Mo *K*α
μ (mm^−1^)	44.78
Crystal size (mm)	0.05 × 0.05 × 0.02

Data collection	
Diffractometer	Bruker D8 Quest
Absorption correction	Multi-scan (*SADABS*; Bruker, 2001 ▸)
*T*_min_, *T*_max_	0.154, 0.511
No. of measured, independent and observed [*I* > 2σ(*I*)] reflections	3493, 191, 190
*R*_int_	0.046
(sin θ/λ)_max_ (Å^−1^)	0.648

Refinement	
*R*[*F* ^2^ > 2σ(*F* ^2^)], *wR*(*F* ^2^), *S*	0.020, 0.052, 1.31
No. of reflections	191
No. of parameters	17
Δρ_max_, Δρ_min_ (e Å^−3^)	1.48, −1.53

Computer programs: *APEX2* and *SAINT* (Bruker, 2004 ▸), *SHELXS* (Sheldrick, 2008 ▸), *SHELXL2014* (Sheldrick 2015 ▸), *DIAMOND* (Brandenburg, 2004 ▸) and *publCIF* (Westrip, 2010 ▸).

## supplementary crystallographic information

### Crystal data

**Table:**

Bi_0.857_Ca_0.143_F_0.143_LaO_0.857_S_2_	*D*_x_ = 6.108 Mg m^−^^3^
*M**_r_* = 404.26	Mo *K*α radiation, λ = 0.71073 Å
Tetragonal, *P*4/*n**m**m*	Cell parameters from 189 reflections
*a* = 4.0548 (9) Å	θ = 4.6–27.4°
*c* = 13.370 (3) Å	µ = 44.78 mm^−^^1^
*V* = 219.82 (11) Å^3^	*T* = 300 K
*Z* = 2	Block, blue
*F*(000) = 342.3	0.05 × 0.05 × 0.02 mm

### Data collection

**Table:**

Bruker D8 Quest diffractometer	*R*_int_ = 0.046
Radiation source: fine-focus sealed tube	θ_max_ = 27.4°, θ_min_ = 4.6°
Profile fitted 2θ/ω scans (Clegg, 1981)	*h* = −5→5
Absorption correction: multi-scan (*SADABS*; Bruker, 2001)	*k* = −5→4
*T*_min_ = 0.154, *T*_max_ = 0.511	*l* = −17→17
3493 measured reflections	3 standard reflections every 90 reflections
191 independent reflections	intensity decay: none
190 reflections with *I* > 2σ(*I*)	

### Refinement

**Table:**

Refinement on *F*^2^	Primary atom site location: difference Fourier map
Least-squares matrix: full	*w* = 1/[σ^2^(*F*_o_^2^) + (0.0318*P*)^2^ + 0.5025*P*] where *P* = (*F*_o_^2^ + 2*F*_c_^2^)/3
*R*[*F*^2^ > 2σ(*F*^2^)] = 0.020	(Δ/σ)_max_ < 0.001
*wR*(*F*^2^) = 0.052	Δρ_max_ = 1.48 e Å^−^^3^
*S* = 1.31	Δρ_min_ = −1.53 e Å^−^^3^
191 reflections	Extinction correction: *SHELXL2014* (Sheldrick, 2015), Fc^*^=kFc[1+0.001xFc^2^λ^3^/sin(2θ)]^-1/4^
17 parameters	Extinction coefficient: 0.033 (3)
0 restraints	

### Special details

**Table:**

Geometry. All esds (except the esd in the dihedral angle between two l.s. planes) are estimated using the full covariance matrix. The cell esds are taken into account individually in the estimation of esds in distances, angles and torsion angles; correlations between esds in cell parameters are only used when they are defined by crystal symmetry. An approximate (isotropic) treatment of cell esds is used for estimating esds involving l.s. planes.

### Fractional atomic coordinates and isotropic or equivalent isotropic displacement parameters (Å^2^)

**Table:**

	*x*	*y*	*z*	*U*_iso_*/*U*_eq_	Occ. (<1)
La1	0.7500	0.7500	0.89827 (6)	0.0127 (3)	
Ca1	0.2500	0.2500	0.62178 (4)	0.0126 (3)	0.143 (4)
Bi1	0.2500	0.2500	0.62178 (4)	0.0126 (3)	0.857 (4)
F1	0.7500	0.2500	1.0000	0.0079 (14)	0.143 (4)
O1	0.7500	0.2500	1.0000	0.0079 (14)	0.857 (4)
S1	0.2500	0.2500	0.8110 (2)	0.0104 (6)	
S2	0.7500	0.7500	0.6221 (3)	0.0236 (8)	

### Atomic displacement parameters (Å^2^)

**Table:**

	*U*^11^	*U*^22^	*U*^33^	*U*^12^	*U*^13^	*U*^23^
La1	0.0115 (4)	0.0115 (4)	0.0151 (5)	0.000	0.000	0.000
Ca1	0.0146 (3)	0.0146 (3)	0.0087 (3)	0.000	0.000	0.000
Bi1	0.0146 (3)	0.0146 (3)	0.0087 (3)	0.000	0.000	0.000
F1	0.0087 (18)	0.0087 (18)	0.006 (3)	0.000	0.000	0.000
O1	0.0087 (18)	0.0087 (18)	0.006 (3)	0.000	0.000	0.000
S1	0.0101 (7)	0.0101 (7)	0.0109 (12)	0.000	0.000	0.000
S2	0.0200 (10)	0.0200 (10)	0.0306 (18)	0.000	0.000	0.000

### Geometric parameters (Å, º)

**Table:**

La1—O1^i^	2.4414 (6)	Ca1—Ca1^v^	4.0548 (9)
La1—O1^ii^	2.4414 (6)	Ca1—Ca1^viii^	4.0548 (9)
La1—F1^i^	2.4414 (6)	Ca1—Bi1^ii^	4.0548 (9)
La1—F1^ii^	2.4414 (6)	Ca1—Bi1^viii^	4.0548 (9)
La1—F1	2.4414 (6)	F1—La1^i^	2.4414 (6)
La1—O1^iii^	2.4414 (6)	F1—La1^viii^	2.4414 (6)
La1—O1	2.4414 (6)	F1—La1^iii^	2.4414 (6)
La1—F1^iii^	2.4414 (6)	O1—La1^i^	2.4414 (6)
La1—S1	3.0954 (13)	O1—La1^viii^	2.4414 (6)
La1—S1^iv^	3.0954 (13)	O1—La1^iii^	2.4414 (6)
La1—S1^ii^	3.0954 (13)	S1—La1^vi^	3.0954 (13)
La1—S1^v^	3.0954 (13)	S1—La1^vii^	3.0954 (13)
Ca1—S1	2.530 (3)	S1—La1^viii^	3.0954 (13)
Ca1—S2	2.8672 (6)	S2—Bi1^iv^	2.8672 (6)
Ca1—S2^vi^	2.8672 (6)	S2—Ca1^iv^	2.8672 (6)
Ca1—S2^vii^	2.8672 (6)	S2—Ca1^v^	2.8672 (6)
Ca1—S2^viii^	2.8672 (6)	S2—Ca1^ii^	2.8672 (6)
Ca1—S2^ix^	3.261 (4)	S2—Bi1^ii^	2.8672 (6)
Ca1—Ca1^ii^	4.0548 (9)	S2—Bi1^v^	2.8672 (6)
Ca1—Ca1^vii^	4.0548 (9)	S2—Ca1^ix^	3.261 (4)
			
O1^i^—La1—O1^ii^	71.917 (18)	S2^viii^—Ca1—Ca1^vii^	135.0
O1^i^—La1—F1^i^	0.0	S2^ix^—Ca1—Ca1^vii^	90.0
O1^ii^—La1—F1^i^	71.917 (18)	Ca1^ii^—Ca1—Ca1^vii^	90.0
O1^i^—La1—F1^ii^	71.917 (18)	S1—Ca1—Ca1^v^	90.0
O1^ii^—La1—F1^ii^	0.0	S2—Ca1—Ca1^v^	45.0
F1^i^—La1—F1^ii^	71.917 (18)	S2^vi^—Ca1—Ca1^v^	135.0
O1^i^—La1—F1	71.917 (18)	S2^vii^—Ca1—Ca1^v^	135.0
O1^ii^—La1—F1	112.29 (4)	S2^viii^—Ca1—Ca1^v^	45.0
F1^i^—La1—F1	71.917 (18)	S2^ix^—Ca1—Ca1^v^	90.0
F1^ii^—La1—F1	112.29 (4)	Ca1^ii^—Ca1—Ca1^v^	90.0
O1^i^—La1—O1^iii^	112.29 (4)	Ca1^vii^—Ca1—Ca1^v^	180.0
O1^ii^—La1—O1^iii^	71.917 (18)	S1—Ca1—Ca1^viii^	90.0
F1^i^—La1—O1^iii^	112.29 (4)	S2—Ca1—Ca1^viii^	135.0
F1^ii^—La1—O1^iii^	71.917 (18)	S2^vi^—Ca1—Ca1^viii^	45.0
F1—La1—O1^iii^	71.917 (18)	S2^vii^—Ca1—Ca1^viii^	135.0
O1^i^—La1—O1	71.917 (18)	S2^viii^—Ca1—Ca1^viii^	45.0
O1^ii^—La1—O1	112.29 (4)	S2^ix^—Ca1—Ca1^viii^	90.0
F1^i^—La1—O1	71.917 (18)	Ca1^ii^—Ca1—Ca1^viii^	180.0
F1^ii^—La1—O1	112.29 (4)	Ca1^vii^—Ca1—Ca1^viii^	90.0
F1—La1—O1	0.0	Ca1^v^—Ca1—Ca1^viii^	90.0
O1^iii^—La1—O1	71.917 (18)	S1—Ca1—Bi1^ii^	90.0
O1^i^—La1—F1^iii^	112.29 (4)	S2—Ca1—Bi1^ii^	45.0
O1^ii^—La1—F1^iii^	71.917 (18)	S2^vi^—Ca1—Bi1^ii^	135.0
F1^i^—La1—F1^iii^	112.29 (4)	S2^vii^—Ca1—Bi1^ii^	45.0
F1^ii^—La1—F1^iii^	71.917 (18)	S2^viii^—Ca1—Bi1^ii^	135.0
F1—La1—F1^iii^	71.917 (18)	S2^ix^—Ca1—Bi1^ii^	90.0
O1^iii^—La1—F1^iii^	0.0	Ca1^ii^—Ca1—Bi1^ii^	0.000 (15)
O1—La1—F1^iii^	71.917 (18)	Ca1^vii^—Ca1—Bi1^ii^	90.0
O1^i^—La1—S1	138.93 (2)	Ca1^v^—Ca1—Bi1^ii^	90.0
O1^ii^—La1—S1	138.93 (3)	Ca1^viii^—Ca1—Bi1^ii^	180.0
F1^i^—La1—S1	138.93 (2)	S1—Ca1—Bi1^viii^	90.0
F1^ii^—La1—S1	138.93 (3)	S2—Ca1—Bi1^viii^	135.0
F1—La1—S1	70.49 (4)	S2^vi^—Ca1—Bi1^viii^	45.0
O1^iii^—La1—S1	70.49 (4)	S2^vii^—Ca1—Bi1^viii^	135.0
O1—La1—S1	70.49 (4)	S2^viii^—Ca1—Bi1^viii^	45.0
F1^iii^—La1—S1	70.49 (4)	S2^ix^—Ca1—Bi1^viii^	90.0
O1^i^—La1—S1^iv^	70.49 (4)	Ca1^ii^—Ca1—Bi1^viii^	180.0
O1^ii^—La1—S1^iv^	70.49 (4)	Ca1^vii^—Ca1—Bi1^viii^	90.0
F1^i^—La1—S1^iv^	70.49 (4)	Ca1^v^—Ca1—Bi1^viii^	90.0
F1^ii^—La1—S1^iv^	70.49 (4)	Ca1^viii^—Ca1—Bi1^viii^	0.000 (15)
F1—La1—S1^iv^	138.93 (3)	Bi1^ii^—Ca1—Bi1^viii^	180.0
O1^iii^—La1—S1^iv^	138.93 (2)	La1^i^—F1—La1^viii^	108.082 (18)
O1—La1—S1^iv^	138.93 (3)	La1^i^—F1—La1^iii^	112.29 (4)
F1^iii^—La1—S1^iv^	138.93 (2)	La1^viii^—F1—La1^iii^	108.082 (18)
S1—La1—S1^iv^	135.72 (11)	La1^i^—F1—La1	108.082 (18)
O1^i^—La1—S1^ii^	138.93 (3)	La1^viii^—F1—La1	112.29 (4)
O1^ii^—La1—S1^ii^	70.49 (4)	La1^iii^—F1—La1	108.082 (18)
F1^i^—La1—S1^ii^	138.93 (3)	La1^i^—O1—La1^viii^	108.082 (18)
F1^ii^—La1—S1^ii^	70.49 (4)	La1^i^—O1—La1^iii^	112.29 (4)
F1—La1—S1^ii^	138.93 (3)	La1^viii^—O1—La1^iii^	108.082 (18)
O1^iii^—La1—S1^ii^	70.49 (4)	La1^i^—O1—La1	108.082 (18)
O1—La1—S1^ii^	138.93 (3)	La1^viii^—O1—La1	112.29 (4)
F1^iii^—La1—S1^ii^	70.49 (4)	La1^iii^—O1—La1	108.082 (18)
S1—La1—S1^ii^	81.84 (4)	Ca1—S1—La1	112.14 (5)
S1^iv^—La1—S1^ii^	81.84 (4)	Ca1—S1—La1^vi^	112.14 (5)
O1^i^—La1—S1^v^	70.49 (4)	La1—S1—La1^vi^	135.72 (11)
O1^ii^—La1—S1^v^	138.93 (3)	Ca1—S1—La1^vii^	112.14 (5)
F1^i^—La1—S1^v^	70.49 (4)	La1—S1—La1^vii^	81.84 (4)
F1^ii^—La1—S1^v^	138.93 (3)	La1^vi^—S1—La1^vii^	81.84 (4)
F1—La1—S1^v^	70.49 (4)	Ca1—S1—La1^viii^	112.14 (5)
O1^iii^—La1—S1^v^	138.93 (3)	La1—S1—La1^viii^	81.84 (4)
O1—La1—S1^v^	70.49 (4)	La1^vi^—S1—La1^viii^	81.84 (4)
F1^iii^—La1—S1^v^	138.93 (3)	La1^vii^—S1—La1^viii^	135.72 (11)
S1—La1—S1^v^	81.84 (4)	Ca1—S2—Bi1^iv^	179.8
S1^iv^—La1—S1^v^	81.84 (4)	Ca1—S2—Ca1^iv^	179.82 (17)
S1^ii^—La1—S1^v^	135.72 (11)	Bi1^iv^—S2—Ca1^iv^	0.0
S1—Ca1—S2	89.91 (9)	Ca1—S2—Ca1^v^	90.0
S1—Ca1—S2^vi^	89.91 (9)	Bi1^iv^—S2—Ca1^v^	90.0
S2—Ca1—S2^vi^	179.82 (17)	Ca1^iv^—S2—Ca1^v^	90.0
S1—Ca1—S2^vii^	89.91 (9)	Ca1—S2—Ca1^ii^	90.0
S2—Ca1—S2^vii^	90.000 (1)	Bi1^iv^—S2—Ca1^ii^	90.0
S2^vi^—Ca1—S2^vii^	90.000 (1)	Ca1^iv^—S2—Ca1^ii^	90.0
S1—Ca1—S2^viii^	89.91 (9)	Ca1^v^—S2—Ca1^ii^	179.82 (17)
S2—Ca1—S2^viii^	90.000 (1)	Ca1—S2—Bi1^ii^	90.0
S2^vi^—Ca1—S2^viii^	90.0	Bi1^iv^—S2—Bi1^ii^	90.0
S2^vii^—Ca1—S2^viii^	179.82 (17)	Ca1^iv^—S2—Bi1^ii^	90.0
S1—Ca1—S2^ix^	180.0	Ca1^v^—S2—Bi1^ii^	179.82 (17)
S2—Ca1—S2^ix^	90.09 (9)	Ca1^ii^—S2—Bi1^ii^	0.00 (2)
S2^vi^—Ca1—S2^ix^	90.09 (9)	Ca1—S2—Bi1^v^	90.0
S2^vii^—Ca1—S2^ix^	90.09 (9)	Bi1^iv^—S2—Bi1^v^	90.0
S2^viii^—Ca1—S2^ix^	90.09 (9)	Ca1^iv^—S2—Bi1^v^	90.0
S1—Ca1—Ca1^ii^	90.0	Ca1^v^—S2—Bi1^v^	0.00 (2)
S2—Ca1—Ca1^ii^	45.0	Ca1^ii^—S2—Bi1^v^	179.82 (17)
S2^vi^—Ca1—Ca1^ii^	135.0	Bi1^ii^—S2—Bi1^v^	179.82 (17)
S2^vii^—Ca1—Ca1^ii^	45.0	Ca1—S2—Ca1^ix^	89.91 (9)
S2^viii^—Ca1—Ca1^ii^	135.0	Bi1^iv^—S2—Ca1^ix^	89.9
S2^ix^—Ca1—Ca1^ii^	90.0	Ca1^iv^—S2—Ca1^ix^	89.91 (9)
S1—Ca1—Ca1^vii^	90.0	Ca1^v^—S2—Ca1^ix^	89.91 (9)
S2—Ca1—Ca1^vii^	135.0	Ca1^ii^—S2—Ca1^ix^	89.91 (9)
S2^vi^—Ca1—Ca1^vii^	45.0	Bi1^ii^—S2—Ca1^ix^	89.9
S2^vii^—Ca1—Ca1^vii^	45.0	Bi1^v^—S2—Ca1^ix^	89.9

Symmetry codes: (i) −*x*+2, −*y*+1, −*z*+2; (ii) *x*, *y*+1, *z*; (iii) −*x*+1, −*y*+1, −*z*+2; (iv) *x*+1, *y*+1, *z*; (v) *x*+1, *y*, *z*; (vi) *x*−1, *y*−1, *z*; (vii) *x*−1, *y*, *z*; (viii) *x*, *y*−1, *z*; (ix) −*x*+1, −*y*+1, −*z*+1.
